# 4-Nitro­phthalamide

**DOI:** 10.1107/S1600536814002955

**Published:** 2014-02-15

**Authors:** Chin Yee Jan, Norzianah Binti Haji Shamsudin, Ai Ling Tan, David J. Young, Seik Weng Ng, Edward R. T. Tiekink

**Affiliations:** a Faculty of Science, Universiti Brunei Darussalam, Jalan Tungku Link BE 1410, Negara Brunei Darussalam; b Department of Chemistry, University of Malaya, 50603 Kuala Lumpur, Malaysia; c Chemistry Department, Faculty of Science, King Abdulaziz University, PO Box 80203 Jeddah, Saudi Arabia

## Abstract

In the title compound, C_8_H_7_N_3_O_4_ (systematic name: 4-nitro­benzene-1,2-dicarboxamide), each of the substituents is twisted out of the plane of the benzene ring to which it is attached [dihedral angles of 11.36 (2)° for the nitro group, and 60.89 (6) and 34.39 (6)° for the amide groups]. The amide groups are orientated to either side of the least-squares plane through the benzene ring with the amine groups being directed furthest apart. In the crystal, a three-dimensional architecture is established by a network of N—H⋯O hydrogen bonds.

## Related literature   

For background to the synthesis of functional phthalocyanines, see: Chin *et al.* (2012 ▶). For the structure of the 1,2-dicarboxamide derivative, see: Hamada *et al.* (2012 ▶). For the synthesis, see: Rasmussen *et al.* (1978 ▶).
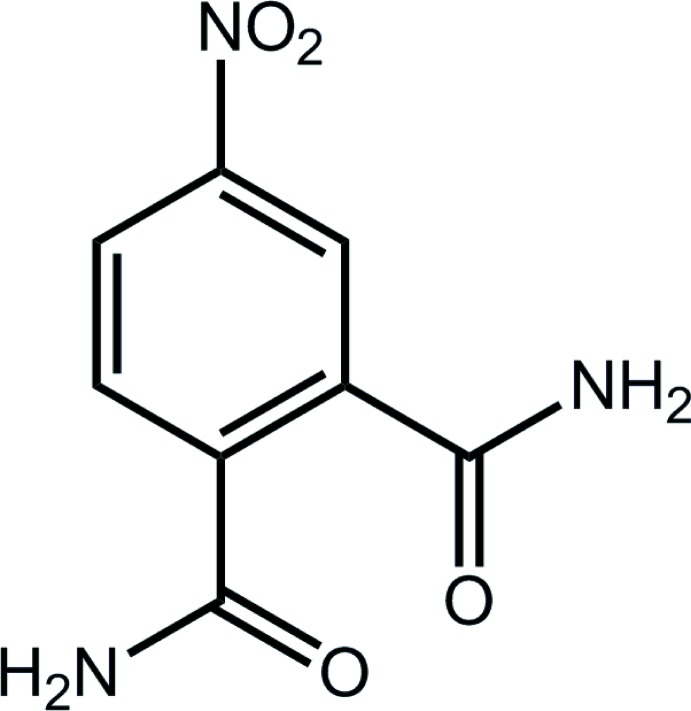


## Experimental   

### 

#### Crystal data  

C_8_H_7_N_3_O_4_
*M* *_r_* = 209.17
Monoclinic, 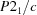

*a* = 7.7425 (2) Å
*b* = 9.6634 (2) Å
*c* = 12.1276 (3) Å
β = 106.008 (3)°
*V* = 872.19 (4) Å^3^
*Z* = 4
Cu *K*α radiation
μ = 1.13 mm^−1^
*T* = 100 K
0.40 × 0.30 × 0.20 mm

#### Data collection  

Agilent SuperNova Dual diffractometer with an Atlas detector
Absorption correction: multi-scan (*CrysAlis PRO*; Agilent, 2013 ▶) *T*_min_ = 0.668, *T*_max_ = 1.000
7908 measured reflections
1821 independent reflections
1748 reflections with *I* > 2σ(*I*)
*R*_int_ = 0.029

#### Refinement  

*R*[*F* ^2^ > 2σ(*F* ^2^)] = 0.032
*wR*(*F* ^2^) = 0.090
*S* = 1.03
1821 reflections
164 parameters
4 restraints
All H-atom parameters refined
Δρ_max_ = 0.33 e Å^−3^
Δρ_min_ = −0.25 e Å^−3^

### 

Data collection: *CrysAlis PRO* (Agilent, 2013 ▶); cell refinement: *CrysAlis PRO*; data reduction: *CrysAlis PRO*; program(s) used to solve structure: *SHELXS97* (Sheldrick, 2008 ▶); program(s) used to refine structure: *SHELXL97* (Sheldrick, 2008 ▶); molecular graphics: *ORTEP-3 for Windows* (Farrugia, 2012 ▶) and *DIAMOND* (Brandenburg, 2006 ▶); software used to prepare material for publication: *publCIF* (Westrip, 2010 ▶).

## Supplementary Material

Crystal structure: contains datablock(s) general, I. DOI: 10.1107/S1600536814002955/hg5382sup1.cif

Structure factors: contains datablock(s) I. DOI: 10.1107/S1600536814002955/hg5382Isup2.hkl

Supporting information file. DOI: 10.1107/S1600536814002955/hg5382Isup3.cml

CCDC reference: 985960

Additional supporting information:  crystallographic information; 3D view; checkCIF report

## Figures and Tables

**Table 1:** Hydrogen-bond geometry (Å, °)

*D*—H⋯*A*	*D*—H	H⋯*A*	*D*⋯*A*	*D*—H⋯*A*
N2—H21⋯O1^i^	0.87 (1)	2.22 (1)	3.0718 (13)	164 (2)
N2—H22⋯O3^ii^	0.88 (1)	2.10 (1)	2.9628 (12)	168 (2)
N3—H31⋯O1^iii^	0.88 (1)	2.42 (1)	3.1288 (13)	138 (1)
N3—H31⋯O3^iv^	0.88 (1)	2.35 (1)	3.0979 (12)	143 (1)
N3—H32⋯O4^v^	0.87 (1)	2.00 (1)	2.8498 (13)	167 (2)

Symmetry codes: (i) 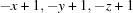; (ii) 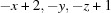; (iii) 
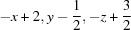
; (iv) 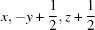; (v) 
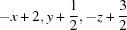
.

**Fig. 1.**
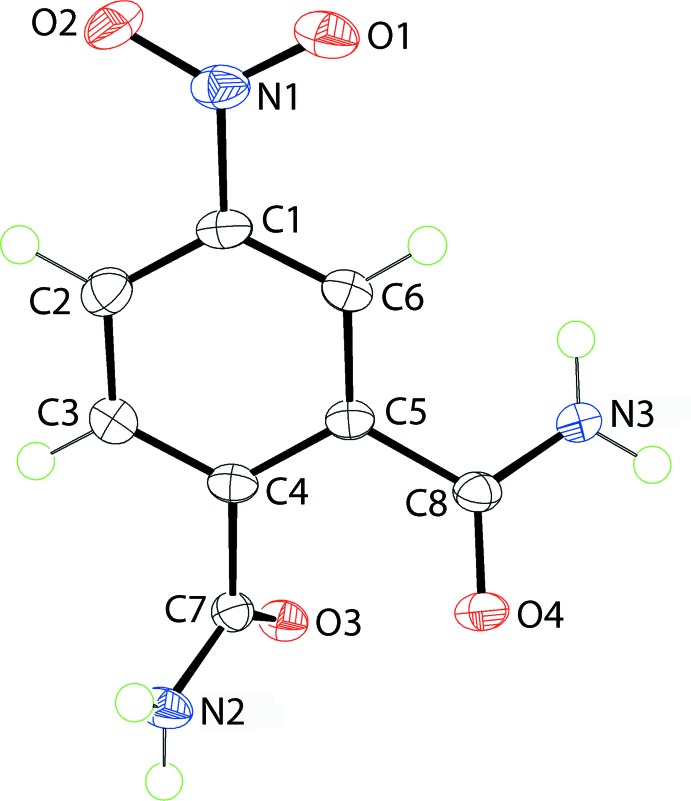
The molecular structure of the title compound showing the atom-labelling scheme and displacement ellipsoids at the 70% probability level.

**Fig. 2.**
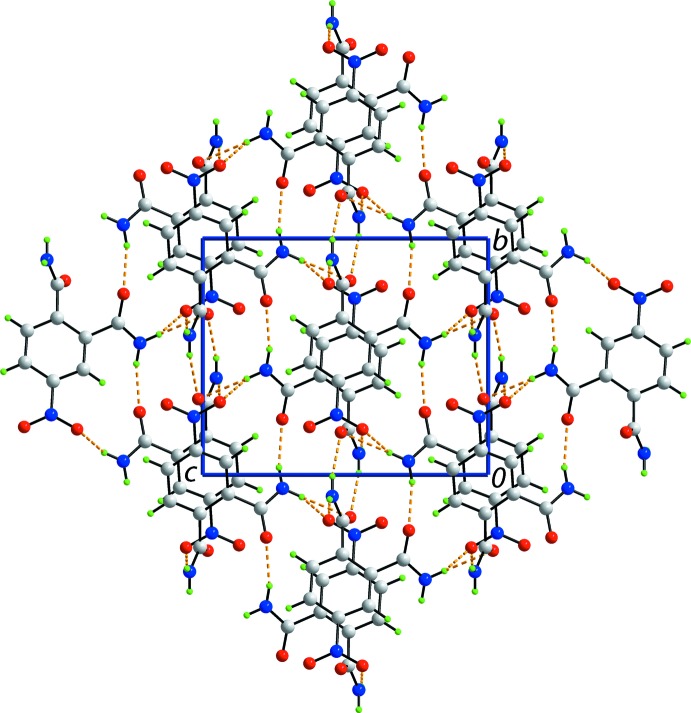
A view of the unit-cell contents of (I) in projection down the a axis. The N—H···O hydrogen bonds are shown as orange dashed lines.

## supplementary crystallographic information

### 2. Structural commentary

As part of our on-going study of functional phthalocyanines, we have previously reported the synthesis and structure of 4-(prop-2-ylnyl­oxy)phthalo­nitrile, prepared from 4-nitro­phthalo­nitrile (Chin *et al.* (2012). The latter, in turn, is prepared by dehydration of the title compound. As the structure of the title compound is not reported, herein its crystal structure determination is described.

In the title compound, Fig. 1, each of the nitro [the O1—N1—C1—C2 torsion angle is 168.48 (10)°], N2-amide [C3—C4—C7—O3 114.92 (12)°] and N3-amide [C6—C5—C8—O4 142.80 (11)°] groups are twisted out of the plane of the benzene ring to which they are attached. The relative orientation of the amide-O atoms places them in positions on either side of the benzene ring, with the amine groups similarly orientated but directed away from each other. As such, there are no intra­molecular hydrogen bonding contacts. Very similar conformations were found for the two independent molecules comprising the asymmetric unit of the 1,2–dicarboxamide parent compound (Hamada *et al.*, 2012).

In the crystal packing, each N—H H atoms forms a N—H···O hydrogen bond with H31 being bifurcated (Table 1); both O1 and O3 accept two hydrogen bonds. The result is a three-dimensional architecture that can be described globally as comprising columns of molecules aligned along the *a* axis (Fig. 2).

### 5. Synthesis and crystallization

The title compound was prepared by modification of a literature procedure (Rasmussen *et al.*, 1978). 4-Nitro­phthalimide and concentrated NH_4_OH were stirred at room temperature for 24 h. The precipitate (an off-white powder) was filtered under vacuum and washed with cold water to provide the title compound in 0.68 g yield (63.8 %). *M*.pt: 465–469 K (literature: 462–464 K). Crystals for the X-ray study were grown from slow evaporation of its aqueous solution.

### 6. Refinement

All C-bound H atoms were refined freely. The N—H atoms were located from difference map and refined with N—H = 0.88±0.01 Å, and with unrestrained *U*_iso_(H).

### Crystal data

**Table:**

C_8_H_7_N_3_O_4_	*F*(000) = 432
*M**_r_* = 209.17	*D*_x_ = 1.593 Mg m^−^^3^
Monoclinic, *P*2_1_/*c*	Cu *K*α radiation, λ = 1.54184 Å
Hall symbol: -P 2ybc	Cell parameters from 4480 reflections
*a* = 7.7425 (2) Å	θ = 3.8–76.2°
*b* = 9.6634 (2) Å	µ = 1.13 mm^−^^1^
*c* = 12.1276 (3) Å	*T* = 100 K
β = 106.008 (3)°	Prism, colourless
*V* = 872.19 (4) Å^3^	0.40 × 0.30 × 0.20 mm
*Z* = 4	

### Data collection

**Table:**

Agilent SuperNova Dual diffractometer with an Atlas detector	1821 independent reflections
Radiation source: SuperNova (Cu) X-ray Source	1748 reflections with *I* > 2σ(*I*)
Mirror monochromator	*R*_int_ = 0.029
Detector resolution: 10.4041 pixels mm^-1^	θ_max_ = 76.4°, θ_min_ = 6.0°
ω scan	*h* = −9→9
Absorption correction: multi-scan (*CrysAlis PRO*; Agilent, 2013)	*k* = −11→12
*T*_min_ = 0.668, *T*_max_ = 1.000	*l* = −14→15
7908 measured reflections	

### Refinement

**Table:**

Refinement on *F*^2^	Primary atom site location: structure-invariant direct methods
Least-squares matrix: full	Secondary atom site location: difference Fourier map
*R*[*F*^2^ > 2σ(*F*^2^)] = 0.032	Hydrogen site location: inferred from neighbouring sites
*wR*(*F*^2^) = 0.090	All H-atom parameters refined
*S* = 1.03	*w* = 1/[σ^2^(*F*_o_^2^) + (0.0552*P*)^2^ + 0.2944*P*] where *P* = (*F*_o_^2^ + 2*F*_c_^2^)/3
1821 reflections	(Δ/σ)_max_ < 0.001
164 parameters	Δρ_max_ = 0.33 e Å^−^^3^
4 restraints	Δρ_min_ = −0.25 e Å^−^^3^

### Special details

**Table:**

Geometry. All e.s.d.'s (except the e.s.d. in the dihedral angle between two l.s. planes) are estimated using the full covariance matrix. The cell e.s.d.'s are taken into account individually in the estimation of e.s.d.'s in distances, angles and torsion angles; correlations between e.s.d.'s in cell parameters are only used when they are defined by crystal symmetry. An approximate (isotropic) treatment of cell e.s.d.'s is used for estimating e.s.d.'s involving l.s. planes.
Refinement. Refinement of *F*^2^ against ALL reflections. The weighted *R*-factor *wR* and goodness of fit *S* are based on *F*^2^, conventional *R*-factors *R* are based on *F*, with *F* set to zero for negative *F*^2^. The threshold expression of *F*^2^ > σ(*F*^2^) is used only for calculating *R*-factors(gt) *etc*. and is not relevant to the choice of reflections for refinement. *R*-factors based on *F*^2^ are statistically about twice as large as those based on *F*, and *R*- factors based on ALL data will be even larger.

### Fractional atomic coordinates and isotropic or equivalent isotropic displacement parameters (Å^2^)

**Table:**

	*x*	*y*	*z*	*U*_iso_*/*U*_eq_	
O1	0.61481 (11)	0.80745 (8)	0.55898 (7)	0.0202 (2)	
O2	0.54207 (13)	0.79742 (9)	0.37347 (8)	0.0266 (2)	
O3	1.04596 (10)	0.17952 (8)	0.51619 (7)	0.0160 (2)	
O4	0.91323 (12)	0.23508 (8)	0.72189 (7)	0.0192 (2)	
N1	0.60572 (12)	0.74606 (10)	0.46837 (8)	0.0177 (2)	
N2	0.76591 (13)	0.09066 (10)	0.45344 (8)	0.0171 (2)	
H21	0.6511 (13)	0.1037 (17)	0.4443 (14)	0.025 (4)*	
H22	0.809 (2)	0.0070 (11)	0.4535 (14)	0.025 (4)*	
N3	1.04060 (14)	0.44255 (10)	0.77908 (8)	0.0182 (2)	
H31	1.096 (2)	0.4063 (17)	0.8462 (10)	0.027 (4)*	
H32	1.050 (2)	0.5306 (10)	0.7667 (13)	0.025 (4)*	
C1	0.67444 (14)	0.60364 (11)	0.47498 (10)	0.0154 (2)	
C2	0.63660 (14)	0.52513 (12)	0.37619 (10)	0.0171 (2)	
H2	0.566 (2)	0.5640 (18)	0.3011 (14)	0.030 (4)*	
C3	0.70007 (15)	0.38986 (12)	0.38425 (10)	0.0164 (2)	
H3	0.677 (2)	0.3367 (15)	0.3164 (13)	0.018 (3)*	
C4	0.80132 (14)	0.33683 (11)	0.48911 (9)	0.0141 (2)	
C5	0.83839 (14)	0.41940 (11)	0.58825 (9)	0.0137 (2)	
C6	0.77318 (15)	0.55445 (11)	0.58101 (10)	0.0150 (2)	
H6	0.795 (2)	0.6102 (16)	0.6473 (13)	0.019 (3)*	
C7	0.88135 (15)	0.19421 (11)	0.49017 (9)	0.0137 (2)	
C8	0.93594 (14)	0.35831 (11)	0.70270 (9)	0.0146 (2)	

### Atomic displacement parameters (Å^2^)

**Table:**

	*U*^11^	*U*^22^	*U*^33^	*U*^12^	*U*^13^	*U*^23^
O1	0.0180 (4)	0.0139 (4)	0.0287 (5)	0.0015 (3)	0.0063 (3)	−0.0027 (3)
O2	0.0284 (5)	0.0171 (4)	0.0269 (5)	0.0051 (3)	−0.0047 (4)	0.0061 (3)
O3	0.0152 (4)	0.0130 (4)	0.0202 (4)	0.0004 (3)	0.0053 (3)	−0.0006 (3)
O4	0.0283 (4)	0.0105 (4)	0.0193 (4)	0.0000 (3)	0.0076 (3)	0.0020 (3)
N1	0.0128 (4)	0.0128 (5)	0.0258 (5)	−0.0002 (3)	0.0024 (4)	0.0013 (4)
N2	0.0153 (5)	0.0111 (5)	0.0246 (5)	0.0009 (4)	0.0049 (4)	−0.0017 (4)
N3	0.0265 (5)	0.0115 (5)	0.0147 (5)	−0.0005 (4)	0.0021 (4)	0.0017 (3)
C1	0.0135 (5)	0.0105 (5)	0.0221 (6)	0.0003 (4)	0.0050 (4)	0.0024 (4)
C2	0.0148 (5)	0.0169 (5)	0.0187 (5)	0.0003 (4)	0.0032 (4)	0.0029 (4)
C3	0.0170 (5)	0.0149 (5)	0.0170 (5)	−0.0009 (4)	0.0045 (4)	−0.0012 (4)
C4	0.0136 (5)	0.0109 (5)	0.0185 (5)	−0.0012 (4)	0.0057 (4)	0.0003 (4)
C5	0.0138 (5)	0.0113 (5)	0.0167 (5)	−0.0008 (4)	0.0053 (4)	0.0012 (4)
C6	0.0157 (5)	0.0118 (5)	0.0180 (5)	−0.0017 (4)	0.0058 (4)	−0.0011 (4)
C7	0.0177 (5)	0.0118 (5)	0.0124 (5)	0.0002 (4)	0.0055 (4)	−0.0002 (4)
C8	0.0177 (5)	0.0112 (5)	0.0161 (5)	0.0018 (4)	0.0069 (4)	−0.0002 (4)

### Geometric parameters (Å, º)

**Table:**

O1—N1	1.2336 (13)	C1—C2	1.3796 (16)
O2—N1	1.2252 (13)	C1—C6	1.3862 (15)
O3—C7	1.2338 (14)	C2—C3	1.3904 (16)
O4—C8	1.2351 (14)	C2—H2	0.997 (16)
N1—C1	1.4698 (14)	C3—C4	1.3945 (15)
N2—C7	1.3338 (14)	C3—H3	0.945 (15)
N2—H21	0.874 (9)	C4—C5	1.4050 (15)
N2—H22	0.875 (9)	C4—C7	1.5097 (14)
N3—C8	1.3280 (15)	C5—C6	1.3934 (15)
N3—H31	0.881 (9)	C5—C8	1.5058 (14)
N3—H32	0.870 (9)	C6—H6	0.943 (16)
			
O2—N1—O1	123.47 (10)	C4—C3—H3	121.1 (9)
O2—N1—C1	118.45 (10)	C3—C4—C5	120.16 (10)
O1—N1—C1	118.09 (9)	C3—C4—C7	118.00 (9)
C7—N2—H21	119.9 (11)	C5—C4—C7	121.64 (9)
C7—N2—H22	118.1 (11)	C6—C5—C4	119.54 (10)
H21—N2—H22	120.6 (15)	C6—C5—C8	120.41 (10)
C8—N3—H31	116.6 (11)	C4—C5—C8	119.89 (9)
C8—N3—H32	122.9 (10)	C1—C6—C5	118.50 (10)
H31—N3—H32	120.4 (15)	C1—C6—H6	121.1 (9)
C2—C1—C6	123.21 (10)	C5—C6—H6	120.4 (9)
C2—C1—N1	118.72 (10)	O3—C7—N2	123.29 (10)
C6—C1—N1	118.06 (10)	O3—C7—C4	119.99 (9)
C1—C2—C3	118.01 (10)	N2—C7—C4	116.51 (9)
C1—C2—H2	121.2 (10)	O4—C8—N3	123.42 (10)
C3—C2—H2	120.8 (10)	O4—C8—C5	119.37 (10)
C2—C3—C4	120.57 (10)	N3—C8—C5	117.20 (9)
C2—C3—H3	118.2 (9)		
			
O2—N1—C1—C2	−11.31 (15)	C2—C1—C6—C5	0.37 (17)
O1—N1—C1—C2	168.48 (10)	N1—C1—C6—C5	179.78 (9)
O2—N1—C1—C6	169.25 (10)	C4—C5—C6—C1	−0.61 (16)
O1—N1—C1—C6	−10.96 (15)	C8—C5—C6—C1	−176.01 (10)
C6—C1—C2—C3	0.44 (17)	C3—C4—C7—O3	114.92 (12)
N1—C1—C2—C3	−178.96 (10)	C5—C4—C7—O3	−60.00 (14)
C1—C2—C3—C4	−1.02 (16)	C3—C4—C7—N2	−60.02 (13)
C2—C3—C4—C5	0.79 (16)	C5—C4—C7—N2	125.05 (11)
C2—C3—C4—C7	−174.21 (10)	C6—C5—C8—O4	142.80 (11)
C3—C4—C5—C6	0.05 (16)	C4—C5—C8—O4	−32.58 (15)
C7—C4—C5—C6	174.86 (9)	C6—C5—C8—N3	−35.96 (14)
C3—C4—C5—C8	175.47 (10)	C4—C5—C8—N3	148.66 (11)
C7—C4—C5—C8	−9.71 (15)		

### Hydrogen-bond geometry (Å, º)

**Table:**

*D*—H···*A*	*D*—H	H···*A*	*D*···*A*	*D*—H···*A*
N2—H21···O1^i^	0.87 (1)	2.22 (1)	3.0718 (13)	164 (2)
N2—H22···O3^ii^	0.88 (1)	2.10 (1)	2.9628 (12)	168 (2)
N3—H31···O1^iii^	0.88 (1)	2.42 (1)	3.1288 (13)	138 (1)
N3—H31···O3^iv^	0.88 (1)	2.35 (1)	3.0979 (12)	143 (1)
N3—H32···O4^v^	0.87 (1)	2.00 (1)	2.8498 (13)	167 (2)

Symmetry codes: (i) −*x*+1, −*y*+1, −*z*+1; (ii) −*x*+2, −*y*, −*z*+1; (iii) −*x*+2, *y*−1/2, −*z*+3/2; (iv) *x*, −*y*+1/2, *z*+1/2; (v) −*x*+2, *y*+1/2, −*z*+3/2.
